# The important ergot alkaloid intermediate chanoclavine-I produced in the yeast *Saccharomyces cerevisiae* by the combined action of EasC and EasE from *Aspergillus japonicus*

**DOI:** 10.1186/s12934-014-0095-2

**Published:** 2014-08-12

**Authors:** Curt AF Nielsen, Christophe Folly, Anaëlle Hatsch, Andrea Molt, Hartwig Schröder, Sarah E O’Connor, Michael Naesby

**Affiliations:** Evolva SA, Duggingerstrasse 23, Reinach, Switzerland; BASF SE, GVF/D - A030, Ludwigshafen, Germany; John Innes Centre, Norwich Research Park, Norwich, UK

**Keywords:** Ergot alkaloids, Chanoclavine-I, Pathway reconstruction, *S. cerevisiae*, Metabolic engineering

## Abstract

**Background:**

Ergot alkaloids are a group of highly bioactive molecules produced by a number of filamentous fungi. These compounds have been intensely studied for decades, mainly due to their deleterious effects in contaminated food and feeds, but also for their beneficial pharmaceutical and agricultural applications. Biosynthesis of ergot alkaloids goes via the common intermediate chanoclavine-I, and studies of the key enzymes, EasE and EasC, involved in chanoclavine-I formation, have relied on gene complementation in fungi, whereas further characterization has been hampered by difficulties of poor EasE protein expression. In order to facilitate the study of ergot alkaloids, and eventually move towards commercial production, the early steps of the biosynthetic pathway were reconstituted in the unicellular yeast *Saccharomyces cerevisiae*.

**Results:**

The genomic sequence from an ergot alkaloid producer, *Aspergillus japonicus*, was used to predict the protein encoding sequences of the early ergot alkaloid pathway genes. These were cloned and expressed in yeast, resulting in *de novo* production of the common intermediate chanoclavine-I. This allowed further characterization of EasE and EasC, and we were able to demonstrate how the N-terminal ER targeting signal of EasE is crucial for activity in yeast. A putative, peroxisomal targeting signal found in EasC was shown to be nonessential. Overexpression of host genes *pdi1* or *ero1*, associated with disulphide bond formation and the ER protein folding machinery, was shown to increase chanoclavine-I production in yeast. This was also the case when overexpressing host *fad1*, known to be involved in co-factor generation.

**Conclusions:**

A thorough understanding of the enzymatic steps involved in ergot alkaloid formation is essential for commercial production and exploitation of this potent compound class. We show here that EasE and EasC are both necessary and sufficient for the production of chanoclavine-I in yeast, and we provide important new information about the involvement of ER and protein folding for proper functional expression of EasE. Moreover, by reconstructing the chanoclavine-I biosynthetic pathway in yeast we demonstrate the advantage and potential of this host, not only as a convenient model system, but also as an alternative cell factory for ergot alkaloid production.

**Electronic supplementary material:**

The online version of this article (doi:10.1186/s12934-014-0095-2) contains supplementary material, which is available to authorized users.

## Background

Ergot alkaloids (EA) belong to a diverse group of natural compounds with a range of biological activities that have important applications in medicine and agriculture [1-3]. Some of these compounds have a notorious neurological effect on humans, possibly due to the structural similarity of these compounds to neurotransmitters like serotonin and dopamine [4]. EAs are produced by a variety of plant associated fungi, mainly of the genera *Claviceps* and *Aspergillus*. The production of ergot alkaloids has been linked to biosynthetic gene clusters found in several species, first in *Claviceps purpurea* [5] and later in *Aspergillus fumigatus* [6,7], *Neotyphodium lolii* [8], and others (see [3] for review).

**Figure 1:**
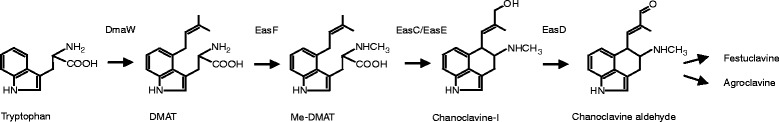
**Biosynthetic pathway to chanoclavine-I and chanoclavine aldehyde.** Ergot alkaloids are synthesized from tryptophan and dimethylallylpyrophosphate (DMAPP, not shown), via dimethylallyltryptophan (DMAT), *N*-methyl-4-dimethylallyltryptophan (Me-DMAT), chanoclavine-I, and chanoclavine aldehyde. The aldehyde is considered to be the branch point in the biosynthetic pathways of different ergot alkaloids, typically via intermediates like agroclavine and festuclavine.

The ergot alkaloids can be divided into three classes, clavines, ergoamides, and ergopeptines, depending on the substitutions found on the basic ergoline scaffold. The biosynthetic pathways leading to various ergot alkaloids have been partially elucidated, and the early steps leading to the common biosynthetic intermediate chanoclavine aldehyde are identical. After biosynthesis of chanoclavine aldehyde the biosynthetic intermediates diverge (Figure 1). Hence, in *C. purpurea* the pathway continues via agroclavine to ergotamine and in *A. fumigatus* via festuclavine to the fumigaclavines [3].

The first step of the common pathway is the electrophilic aromatic addition of dimethylallyl-pyrophosphate (DMAPP) to the 4 position of tryptophan to form dimethylallyl-tryptophan (DMAT). The reaction is catalysed by a prenyl transferase, DmaW (fgaPT2 in *A. fumigatus*) [6,9]. The second step, the methylation of DMAT to form *N*-methyl-4-dimethylallyltryptophan (Me-DMAT), is catalysed by the methyltransferase EasF (fgaMT in *A. fumigatus*) [10].

While the biosynthesis of Me-DMAT is well understood, the mechanistic basis behind the conversion of Me-DMAT to chanoclavine-I is less clear. The conversion of Me-DMAT to chanoclavine-I was investigated by gene disruption and complementation studies in *C. pupurea* and *A. fumigatus*: Lorenz and co-workers [11] used a mutated *C. purpurea* strain P1 to show that deletion of the *easE_Cp* (*ccsA*) gene abolished production of chanoclavine-I and any downstream products. Instead an accumulation of Me-DMAT was seen, indicating a block in the pathway after this intermediate. Alkaloid biosynthesis could be restored by expressing a GFP fusion construct of the *easE_Cp* gene. Analogously, Goetz and co-workers [12] disrupted the *easC_Af* gene in *A. fumigatus*, and also observed accumulation of Me-DMAT and the absence of downstream products. Furthermore, a similar pattern was observed when *easE_Af* was disrupted, in concordance with the *C. purpurea* results (above). In both of the *easE* and *easC* deletion strains, the alkaloid pathway could be restored by re-introduction of the corresponding wild type allele. Most recently Ryan and co-workers [13] transferred part of the *A. fumigatus* EA cluster, comprising the four genes *dmaW*, *easF*, *easC*, and *easE*, into *A. nidulans*, a fungus known as a non-producer of any EA. This partial cluster conferred the ability to produce chanoclavine-I, further suggesting that EasE and EasC are sufficient for the conversion from Me-DMAT. However, the involvement of enzymes from cryptic EA clusters in *A. nidulans* cannot be excluded [14].

Interestingly, EasC_Af contains a C-terminal amino acid motif (SRL), which is a classic type 1 peroxisomal targeting signal (PTS1) [15]. Inspection of related published sequences reveals that similar PTS1 signals are found in many EasC homologues from *Aspergillus spp*., but not from *Claviceps spp*. EasE_Af, on the other hand, appears to have an N-terminal signal peptide for the secretory pathway, as previously suggested [12]. The sequence has the typical core of hydrophobic amino acids within the first 15–30 residues, and a signal peptide is predicted by the SignalP model [16]. However, the implication of these localisation signals remains unclear, particularly in light of the apparent co-operation between EasC and EasE. The EasC proteins have similarity to peroxisomal catalases [6,7,17]. EasC_Af (the *easC_Af* gene product) was purified after expression in *E. coli*, and *in vitro* catalase activity of EasC_Af was shown using H_2_O_2_ as substrate [12]. However, when the enzyme was incubated with Me-DMAT, no new product was detected. Extensive efforts were made to produce active EasE_Af from *E. coli* or *S. cerevisiae* [12]. However, in all cases, incubation of EasE with Me-DMAT, or of EasE with Me-DMAT and EasC, failed to produce any new product. Therefore, biochemical studies to understand the transformation of Me-DMAT to chanoclavine-I have not been possible.

As an alternative approach to gain information about the early EA pathway we undertook the reconstitution of the chanoclavine-I pathway in yeast (*S. cerevisiae*). Yeast is the work horse of eukaryotic gene expression and is easily amenable to genetic manipulation [18,19]. Heterologous genes and proteins are generally well expressed, and yeast has a long history of use for industrial production of a variety of products [20,21]. We report here the successful engineering of yeast for *de novo* production of chanoclavine-I, overcoming the previously reported difficulties of expressing EasE. Further, we provide new insight into the roles of EasC and EasE in the intriguing biochemical conversion from Me-DMAT into chanoclavine-I, the key step in the early ergot alkaloid pathway.

## Results

### Prediction of dmaW coding sequences

An ergot alkaloid gene cluster was recently identified in *A. japonicus* [22], one of several fungal species currently being investigated for their capacity to produce molecules of potential commercial importance [23-25]*. A. japonicus* was previously reported to produce cycloclavine [26], an EA of the clavine group, and the genome sequence of the cluster displays high homology to EA clusters of *Claviceps spp.* and *Aspergillus spp.* This cluster therefore provided an interesting alternative for studying the chanoclavine-I pathway. The putative coding sequences (CDS) of *dmaW*, *easF*, *easC*, and *easE* were predicted, using free online gene prediction software and by alignment to homologues in the GenBank database (http://blast.ncbi.nlm.nih.gov/Blast.cgi). Analysis of the *dmaW* sequence in the *A. japonicus* genome revealed two different CDS predictions: one prediction was for a single open reading frame of 1602 bps, corresponding to a 534 amino acid protein, whereas another prediction, with the same translation start codon, was for two exons of 1287 bps and 150 bps separated by an 85 bps intron, corresponding to a 479 amino acid protein. Both of these predictions were slightly longer than similar DmaW enzymes found in the GenBank database, where the lengths of DmaW homologues were in the range of 435–465 amino acids. Moreover, a multiple protein alignment in GenBank, using either of the two *A. japonicus* DmaW predictions as query, showed a lack of homology beyond approx. 428 amino acids, i.e. beyond the predicted first exon. A somewhat similar situation was seen for the two entries of *A. fumigatus* DmaW (Additional file 1: Figure S1). Hence, we decided to test, not only the two predictions encoding 534 amino acids (*dmaW_Aj1*) and 479 amino acids (*dmaW_Aj2*), but also a shorter version encoding 429 amino acids (*dmaW_Aj3*), corresponding to the predicted first exon. For *easF*, representing the next step in the pathway, we tested the homologues *easF_Af* and *easF_Aj*, from *A. fumigatus* and *A. japonicus,* respectively.

### Me-DMAT production in yeast

**Figure 2:**
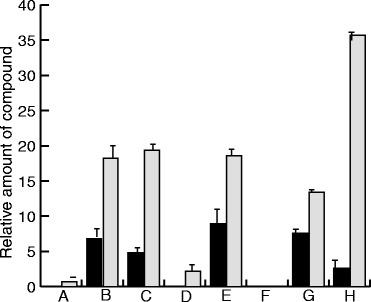
**Activity of different DmaW and EasF enzymes.** Relative production of DMAT (black bars) and Me-DMAT (grey bars) were analysed in strains co-expressing EasF_Aj in combination with DmaW_Aj1 **(A)**, DmaW_Aj2 **(B)**, DmaW_Aj3 **(C)**, DmaW_Af **(D)**, or DmaW_Cp **(E)**. Expression of DmaW_Aj1 and DmaW_Af resulted in only small amounts of compounds compared to the other three DmaW homologues. Similarly, DmaW_Aj2 was co-expressed with the two homologues EasF_Aj **(G)** or EasF_Af **(H)** and relative DMAT and Me-DMAT amounts were measured. The EasF_Af resulted in more than double the amount of Me-DMAT compared to EasF_Aj. The control strain **(F)** carried an empty plasmid with no DmaW expression. The vertical axis shows arbitrary units based on area under the HPLC peak.

To analyse the biosynthesis of chanoclavine-I we first constructed a yeast strain designed to produce the chanoclavine-I precursor Me-DMAT. For this, plasmids were constructed with synthetic, yeast codon optimized genes corresponding to each of the three *dmaW* predictions described above, as well as the *dmaW_Cp* [GenBank:AJ011963] and *dmaW_Af* [GenBank:XM_751048] (Table 1). All genes were tested by co-expression with a codon optimized *easF_Aj* gene. The *easF_Aj* gene was cloned in pRS315-C/A, and each *dmaW* gene was cloned in the pRS316-C/A, allowing expression from the yeast Cup1 promoter. Combinations of *easF_Aj* and each of the *dmaW* genes were then introduced in yeast and expressed by inducing the Cup1 promoter. After 72 h of growth at 30°C, the culture supernatants were analysed by LC-MS, and the expected mass to charge ratio (m/z) of DMAT (m/z = 273.159 +/− 0.01) and Me-DMAT (m/z = 287.175 +/− 0.01) were extracted from the total ion chromatograms. The area under the corresponding peaks was calculated and compared, indicating production of both DMAT and Me-DMAT in the strains expressing *dmaW_Aj2*, *dmaW_Aj3*, and *dmaW_Cp*, but essentially no production with *dmaW_Aj1* or *dmaW_Af* (Figure 2). We suspect that *dmaW_Aj1* is likely to be an incorrect CDS prediction. We also noted that, compared to the homologue used in a previous study [6], *dmaW_Af* has a 24 bp deletion and speculate that the sequence of *dmaW_Af* may also be an incorrect prediction. (Additional file 1: Figure S1). Inspection of the total ion current chromatogram revealed the appearance of several additional minor peaks in strains accumulating the putative DMAT and Me-DMAT. These were not analysed further but we speculate that they could be the result of non-enzymatic conversions due to low pH in the culture medium in the late stationary phase (pH < 4 after 72 hours). Alternatively, they could be shunt products resulting from the activity of yeast metabolic enzymes. In any case, the apparent accumulation of DMAT suggested a relatively poor activity of the methyl transferase EasF_Aj. In a separate experiment we therefore tested the *A. fumigatus* homologue EasF_Af in comparison to EasF_Aj. Again, the combination of DmaW_Aj2 and EasF_Aj resulted in accumulation of DMAT (Figure 2,G) although the relative levels of DMAT and Me-DMAT showed some variation compared to the previous experiment (Figure 2,B). This could be due to some *in vivo* instability of the compounds, as indicated by the shunt product formation mentioned above, but since it was not the focus of this work it was not investigated further. More importantly, when combining DmaW_Aj2 with the *A. fumigatus* homologue EasF_Af it appeared that much more of the produced DMAT was converted to Me-DMAT (Figure 2,H). Hence, for further studies the combination of *dmaW_Aj2* and *easF_Af*, which showed the highest production of Me-DMAT, was integrated into the yeast genome. The new strain was used to purify Me-DMAT, and the compound was analysed by ^1^NMR to confirm the compound identity (Additional file 1: Figure S2a).

**Table 1:** **Genes used in this study**

**CDS name**	**Source**	**Amino acids**
***dmaW_Aj1***	WO2012/116935	534
***dmaW_Aj2***	WO2012/116935	479
***dmaW_Aj3***	WO2012/116935	428
***dmaW_Af***	XM_751048	451
***dmaW_Cp***	AJ011963	448
***easF_Aj***	WO2012/116935	340
***easF_Af***	XM_751050	339
***easC_Aj***	WO2012/116935	510
***easC_Af***	XM_751047	520
***easC_Aj -PTS1***	WO2012/116935	507
***easE_Aj***	WO2012/116935	622
***easE_Af***	XM_751049	628
***easE_Cp***	AJ011965	483
***easE_Aj -N sig.***	WO2012/116935	594
***pdi1/easE_Aj***	D00842/WO2012/116935	615
***pdi1_Sc***	D00842	522
***ero1_Sc***	NM_001182493	563
***fad1_Sc***	NM_001180104	306

Gene names are followed by a two-letter code to indicate species of origin: Aj: *A. japonicus*; Af: *A. fumigatus*; Cp: *C. purpurea*; Sc: *S. cerevisiae*. All genes were synthesized with yeast codon optimization, except for *pdi1_Sc*, *ero1_Sc*, and *fad1_Sc*, which were prepared by PCR on yeast genomic DNA. Synthesis and PCR primer design was based on the named sources to give proteins of the indicated length.

### Chanoclavine-I production in yeast

**Figure 3:**
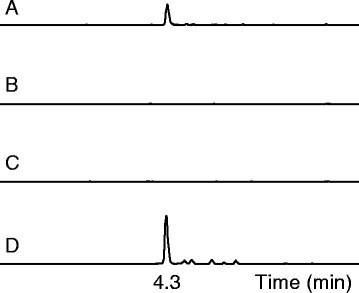
**Production of chanoclavine-I enabled by EasE from** ***A. japonicus*** **.** Yeast strains co-expressing DmaW_Aj2, EasF_Af, and EasC_Aj in combination with one of three different homologues, EasE_Aj **(A)**, EasE_Af **(B)**, or EasE_Cp **(C)**, were analysed by UPLC-TOF for chanoclavine-I production. Only the strain co-expressing EasE_Aj produced chanoclavine-I, whereas no chanoclavine-I was detected in strains expressing either EasE_Af or EasE_Cp. Retention time and m/z of **(A)** corresponded to a chanoclavine-I reference extracted from *A. japonicus* mycelium **(D)**.

Having established the production of Me-DMAT in yeast, we next addressed the conversion of this compound into chanoclavine-I. Preliminary results in our laboratory had shown poor expression of a C-terminal GFP-fusion construct of EasE_Aj, which is consistent with the reported difficulties of purifying the corresponding enzyme, EasE_Af, from *A. fumigatus* [12]. Therefore, in this study, we tested several *easE* homologues from *A. japonicus*, *A. fumigatus*, as well as from *C. purpurea*. To investigate the hypothesis that EasC and EasE are both required for this conversion [12,13] we expressed *easC_Aj*, in combination with each of the three different *easE* homologues, in the background of the Me-DMAT producing yeast (see above). Each *easE* homologue was cloned into pRS313-G/C, and *easC_Aj* into pRS315-P/A. When combining *easC_Aj* and *easE_Aj* we saw the appearance of a new compound with a retention time of 4.3 min, which had the expected m/z of chanoclavine-I (m/z = 257.165 +/− 0.01). The compound co-eluted with a compound of identical mass found in an *A. japonicus* mycelium extract (Figure 3). The compound at 4.3 min was purified, and the identity was confirmed by ^1^H NMR to be chanoclavine-I (Additional file 1: Figure S2b). Chanoclavine-I was not detected in strains expressing *easC_Aj* in combination with either *easE_Af* or *easE_Cp*, indicating that the *A. fumigatus* and *C. purpurea* homologues used in this study may not produce functional enzymes in yeast. In strains expressing only an *easE* or an *easC* homologue no chanoclavine-I was detected, supporting the hypothesis that both EasE and EasC are required for its biosynthesis. The combination of *easC_Aj* and *easE_Aj* was integrated into the genome of the Me-DMAT producing strain (see above), and the resulting strain was used to purify chanoclavine-I, which was again characterized by ^1^H NMR.

### Peroxisomal targeting signal is not needed in yeast

**Figure 4:**
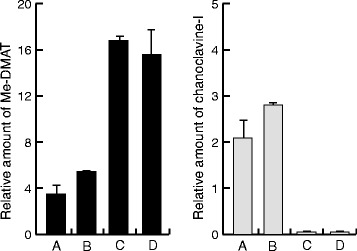
**Roles of EasE signal peptide, and EasC tripeptide ARL.** Relative production of Me-DMAT (left panel) and chanoclavine-I (right panel) was analysed, of a Me-DMAT producing strain, co-expressing EasC and EasE proteins with different localization signals. Wild type EasE_Aj was co-expressed in combination with either wt EasC_Aj **(A)** or EasC_Aj -PTS1which has no PTS1 **(B)**. Removal of the PTS1 tripeptide ARL resulted in a slight increase of Me-DMAT and chanoclavine-I. However, when an N-terminally truncated EasE_Aj –N sig. was co-expressed with either wt EasC_Aj **(C)** or EasC_Aj -PTS1 **(D)**, production of chanoclavine-I was essentially abolished. The loss of function, due to the N-terminal truncation of EasE_Aj, resulted in increased accumulation of the precursor Me-DMAT (**C and D** compared to **A and B**). The vertical axes show arbitrary units based on areas under the HPLC peaks.

As noted by Goetz and co-workers [12] EasC_Af has a classical PTS1 peroxisomal targeting sequence, the tri-peptide SRL, at its carboxy-terminal end similar to PTS1 signals commonly found in yeast [15]. One such putative signal, ARL, is also present in the *A. japonicus* EasC_Aj sequence, as well as in other homologues within the *Aspergillus* genus. However, in the *Claviceps* genus no obvious PTS1 signal is found and the *C. purpurea* EasC enzyme instead has a C-terminal IVE tri-peptide, which has no resemblance to the PTS1 consensus sequence. Assuming the EA enzymes from these fungi have similar function, and therefore localization, this is somewhat puzzling and we therefore wondered if the PTS1 of *Aspergilli* is important for function. Hence, we prepared an EasC-Aj version in which the ARL triplet was deleted. A set of strains were prepared expressing the integrated *dmaW_Aj2* and *easF_Af*, together with *easE_Aj* (in pRS313-G/C) and either the new *easC-Aj -PTS1* version (in pRS315-P/A) or the original full length *easC_Aj* (in pRS315-P/A). To our surprise we saw no major difference in the ability to produce Me-DMAT and chanoclavine-I. In fact, the version without the putative PTS1 sequence resulted in slightly higher concentrations of Me-DMAT and chanoclavine-I (Figure 4)

.

### N-terminal signal is crucial for EasE function

A common feature observed for EasE enzymes is a high number of hydrophobic amino acids at the N-terminal end, which is typically associated with signal peptides for the secretory pathway. Analysis of the amino acid sequence of EasE_Aj, using the online SignalP server [16], confirmed this notion by predicting a signal peptide. A putative cleavage site in EasE_Aj was predicted after pos. 29, i.e. between A and V. We used this information to prepare a truncated version of the enzyme, which lacked the N-terminal sequence, and expressed the corresponding gene from the pRS313-G/C plasmid, in the Me-DMAT producing strain, together with *easC_Aj* (in pRS315-P/A). The N-truncation seriously impaired the functional expression of EasE_Aj, and essentially no chanoclavine-I was detected using this enzyme. Instead, accumulation of the precursor compound Me-DMAT was observed (Figure 4).

**Figure 5:**
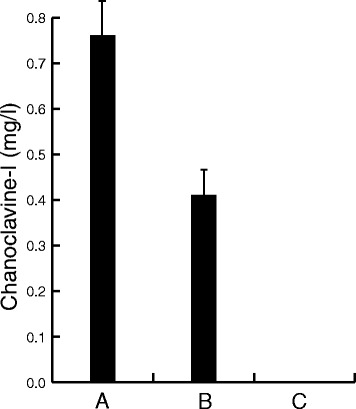
**Signal peptide from Pdi1 partially restores EasE function.** Concentration (mg/l) of chanoclavine-I, was measured in the growth medium, of a strain expressing DmaW_Aj2, EasF_Af, and EasC_Aj after co-expression of different versions of EasE. Expression of the wt EasE_Aj **(A)** resulted in production of 0.75 mg/l chanoclavine-I, whereas expression of a modified version **(B)**, comprising the N-terminal signal peptide from Pdi1, resulted in production of approximately half of this amount. Complete deletion of the N-terminal sequence **(C)** abolished the function of EasE_Aj, and essentially no chanoclavine-I was detected.

To further evaluate the function of the putative signal peptide we prepared a version of EasE_Aj, in which we replaced the N-terminal 31 amino acids (predicted signal peptide including the cleavage site) with the known signal peptide from the native yeast Pdi1 protein. DNA sequences were fused to encode the N-terminal 24 amino acids from Pdi1, which includes the cleavage site, followed by the EasE_Aj peptide from pos. 32 (Additional file 1: Table S2). The fusion protein was tested as described for the truncated version. When expression was compared to the original EasE_Aj approximately half the chanoclavine-I production level was observed, indicating that the Pdi1 signal peptide provided functionality to the enzyme (Figure 5). This was taken as an indication that for proper function the EasE needs to enter the secretory pathway. It is not clear why the yields were lower when the Pdi1 sequence was used but possibly the cleavage site of the EasE_Aj could have been wrongly predicted and proper cleavage thus affected. Translocation across the ER membrane is a complex matter [27], but presumably a lack of cleavage would cause proteins to getting stuck in the membrane, and/or to being rapidly degraded, but this was not investigated. Testing alternative fusions of EasE_Aj to the Pdi1 signal, or fusions to other known signal peptides, might also shed more light on the function and requirements of the N-terminal signal sequence of EasE_Aj.

### Pdi1 or Ero1 overexpression improves the activity of EasE in yeast

**Figure 6:**
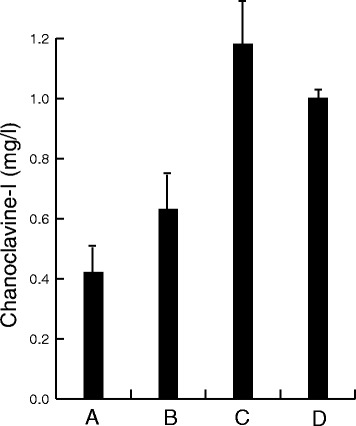
**Effects of host engineering on chanoclavine-I production.** Concentration (mg/l) of chanoclavine-I, was measured in the growth medium, of a chanoclavine-I producing control strain **(A)** and after over-expression of Pdi1 **(B)**, Ero1**(C)**, or Fad1 **(D)**. Over-expression of these native yeast genes all resulted in an increased production of chanoclavine-I, relative to the control. All strains expressed the integrated, heterologous pathway to chanoclavine-I.

We speculated that the importance of the N-terminal sequence is linked to a requirement for disulphide bond formation as part of the protein maturation process, which would happen during ER associated translation. EasE_Aj has a total of 9 cysteines which could potentially form disulphide bridges, and the majority of these cysteines are highly conserved among EasE enzymes (Additional file 1: Figure S3). For a preliminary evaluation of the importance of these cysteines we mutated the first seven of these individually, replacing them with alanine. All of these mutations resulted in complete loss of function (data not shown). The native yeast enzymes Pdi1 and Ero1 are known to have a function in the formation of disulphide bonds in ER (see e.g. [28] for review). Hence, a pair of chanoclavine-I producing strains were prepared in which we over-expressed one of the two genes, either *pdi1* or *ero1*, to test whether this would have any impact on the production of chanoclavine-I. Over-expression of *pdi1* (in pRS313-G/C) resulted in an approx. 50% increase in chanoclavine-I production, whereas *ero1* over-expression (in pRS313-C/A) caused an almost 3 fold increase (Figure 6), supporting a possible involvement of disulphide bond formation in proper EasE folding. However, all chanoclavine-I producing strains also accumulated substantial amounts of Me-DMAT, indicating continued limitations in the conversion of Me-DMAT to chanoclavine-I.

### Fad1 overexpression improves the activity of EasE in yeast

EasE has previously been described as a flavin adenine dinucleotide (FAD) dependent reductase or dehydrogenase, and in support of this the structural similarity to the class of berberine bridge enzymes (BBE) has previously been pointed out [11]. In plants the BBE enzyme is involved in alkaloid biosynthesis, and it has been associated with transport from ER to the vacuole [29]. BBE was shown to bi-covalently bind a flavin co-factor [30] and the binding site, involving a histidine and a cysteine, seems to be conserved in EasE_Aj and other EasE enzymes (Additional file 1: Figure S4). With ample evidence that EasE is FAD dependent, we speculated whether an increased supply of this co-factor would directly improve the activity of EasE. An attempt to increase the supply via the growth medium showed no effect on chanoclavine-I production (data not shown), so instead we cloned the native yeast *fad1* gene, which encodes the enzyme responsible for synthesis of FAD from flavin mono nucleotide (FMN). Over-expression of this gene, in a strain harbouring the chanoclavine-I pathway, led to a 2.5 fold increase in chanoclavine-I production (Figure 6) which we interpreted as an effect of improved co-factor supply.

## Discussion

Heterologous expression in yeast of the four enzymes DmaW_Aj2, EasF_Af, EasC_Aj, and EasE_Aj led to production of chanoclavine-I, demonstrating that yeast is a suitable host for producing ergot alkaloids. This work also confirms, in accordance with previous published studies, that EasC and EasE are solely responsible for the conversion of Me-DMAT to chanoclavine-I without the involvement of other EA enzymes. Secondary metabolism in *S. cerevisiae* is quite limited and it seems unlikely that any yeast enzyme would be involved in the highly specialized metabolic process of ergot alkaloid biosynthesis. The production of chanoclavine-I depended on the expression of EasE from *A. japonicus*, whereas EasE from *A. fumigatus* or *C. purpurea* were not active when expressed in yeast. We speculate that the predicted coding regions of *easE_Af* and *easE_Cp*, along with *dmaW_Aj3*, could be incorrect (see below, and Additional file 1: Figure S3 and S5). Certainly, the prediction of intron and exon sequences in filamentous fungi is still a challenge.

Our results support the notion that EasE and EasC co-operate to produce chanoclavine-I, but it is still not clear how or where this process occurs. Knockout experiments of easC and easE in *Aspergillus fumigatus* [12], along with the data presented here, indicate that the catalytic activities of two classes of enzymes, a catalase encoded by easC and a FAD-dependent oxidoreductase encoded by easE, are both required to convert Me-DMAT to chanoclavine-I. Deletion mutants of easC and easE accumulate Me-DMAT, which suggests, though does not prove, that these two enzymes act in a coordinated fashion. It is also possible that the ergot alkaloid pathway requires disproportionation of H_2_O_2_, and that the function of EasC is to remove H_2_O_2_ derived from the oxidative activities of EasE. More extensive biochemical experiments with active EasE enzyme would be able to shed further light on these questions.

As shown here the putative PTS1 signal of EasC_Aj was not essential for function, and it is possible that even with the PTS1 signal the natural cellular distribution involves multiple locations, as seen for some native yeast proteins [31,32]. The N-terminal signal of the EasE, on the other hand, was clearly crucial for function, and the fact that a native yeast signal peptide from Pdi1 partially restored function indicates that EasE contains a genuine ER targeting signal. This would suggest that folding and disulphide bond formation in the ER is needed for proper maturation of EasE and that it passes through the secretory pathway before, possibly, joining up with EasC. Cellular localization studies might help elucidate this puzzle, just as further progress toward obtaining *in vitro* activity of EasC and EasE should allow us to explore the interdependence of these two enzymes on one another.

The case for oxidative folding of EasE in ER is supported by the observed increase in chanoclavine-I production after over-expression of either of the two key enzymes in the yeast disulphide bridge formation machinery. Ero1 is a sulfhydryl oxidase responsible for generating disulphide bonds that are passed on to Pdi1, which in turn oxidizes the cysteines of newly translated proteins [28,33,34]. The EasE family contains several highly conserved cysteines that would be available for oxidation, although one cysteine is likely to be involved in binding the FAD co-factor.

Interestingly, a multiple sequence alignment of the EasE_Aj and its closest homologues showed some unexpected dissimilarity regarding EasE_Af and EasE_Cp (Additional file 1: Figure S5). The first approx. 130 amino acids of EasE_Af showed no similarity to the consensus sequence, whereas EasE_Cp seemed to be completely lacking the N-terminal domain. An earlier prediction of EasE_Cp [11] suggested a 483 amino acid peptide derived from two exons. A newer GenBank entry [GenBank:JN186799], however, predicts a third exon at the 5-end and encodes a 595 amino acid protein, including a signal peptide as predicted by the SignalP model [16]. The study [11] reported complementation of an *easE_Cp* gene knock-out after integration of a PCR fragment comprising the same *easE* gene including its native promoter region. We speculate that this PCR fragment may by chance have included the first exon, allowing correct splicing and, hence, full complementation. Complementation by re-integration into the *easE* locus was excluded, since integration at the *niaD* locus was confirmed by Southern hybridization. A similar approach was used for studying the *easE* gene in *A. fumigatus* [12]. The native *easE* gene was first disrupted, which abolished function, and the WT sequence was then re-integrated along with 1445 bps 5’-flanking sequence. The inclusion of upstream sequence would allow the fungus to splice the gene correctly, and any divergence between the mature mRNA and the predicted CDS of *easE_Af* would not have been detected. We analysed the genomic region of *A. fumigatus* chromosome II using an online intron prediction model, which suggested an 1809 bps *easE_Af* coding sequence, encoding a protein of 602 amino acids including a putative N-terminal signal peptide. This newly predicted protein shows very high sequence similarity to other EasE proteins in the multiple alignment (Additional file 1: Figure S3). We synthesized a yeast codon optimized version of this predicted easE_Af, and tested its ability to support chanoclavine-I formation. In a strain co-expressing dmaW_Cp, easF_Af, and easC_Aj, the expression of either easE_Af or easE_Aj resulted in similar levels of chanoclavine-I production (data not shown). This would indicate that, at least in this case, the EasC and EasE enzymes from different fungi are capable of working together. However, further studies are needed to confirm the results, and to investigate the nature of co-operation between EasC and EasE.

## Conclusion

We show here that EasC and EasE are responsible for the conversion of Me-DMAT to chanoclavine-I in yeast. Although the activity of these enzymes appears to be linked, the cellular localization of the enzymatic reaction is unclear. The N-terminal peptide sequence of EasE was identified as a signal for the secretory pathway, and a role for ER-associated folding was supported by a beneficial effect of overexpressing host enzymes involved in protein disulphide bond formation.

Surprisingly, a putative C-terminal PTS1 in EasC appeared to be non-essential, at least in a yeast host background, and the cellular localization of EasC cannot be predicted. Further studies will be needed to address questions regarding cellular localization of the enzymes, and much work is still pending towards a full understanding of the biochemical reactions involved. Nonetheless, the knowledge we have obtained in this study should enable further elucidation of these reactions.

Importantly, we also demonstrate that the biosynthetic pathway for chanoclavine-I, the central biosynthetic precursor for all ergot alkaloids, can be transferred to the industrially important host, *S. cerevisae.* This discovery will greatly facilitate further genetic and metabolic engineering of the ergot alkaloid pathway, which will potentially have a broad range of pharmaceutical and agrochemical applications. Our results therefore provide an entry point on the road to commercial scale production of known or novel ergot alkaloids with important industrial applications.

## Methods

### DNA and protein sequence analysis

Computer-aided sequence analysis was done using Vector NTI 9.1.0 software (Invitrogen Corp. 2004) and the free online software FGENESH (http://linux1.softberry.com/berry.phtml), GENSCAN (http://genes.mit.edu/GENSCAN.html), and the NCBI server (http://www.ncbi.nlm.nih.gov). Signal peptides were predicted using the SignalP 4.0 tool [16].

### Preparation and cloning of genes in yeast expression vectors

Synthetic genes, codon optimized for expression in yeast, were manufactured by DNA2.0 Inc., Menlo Park, CA, USA or GeneArt AG, Regensburg, Germany. All sequences were derived from *A. japonicus*, *A. fumigatus*, or *C. purpurea*. The genes encode the amino acid sequences, plus a translation stop codon, of DmaW_Af [GenBank: XP_756141], DmaW_Cp [GenBank:CAB39314], EasF_Af [GenBank:XP756143], EasC_Af [GenBank:XP756140], EasE_Af [GenBank:XP756142], and EasE_Cp [GenBank:CAB39328]. The sequences *dmaW_Aj1*, *easF_Aj*, *easC_Aj*, and *easE_Aj* were predicted, based on the genomic DNA sequence of the cycloclavine gene cluster of *A. japonicus* [22]. All genes were synthesized with the DNA sequence AAGCTTAAA, containing a *Hind*III restriction recognition site, at the 5’-end and with a *Sac*II recognition site at the 3’-end (CCGCGG), and these sites were used for cloning. All PCR primers used for sub-cloning contained these sequences. Standard PCR conditions were used according to manufacturer’s recommendations (BioRad iProof High Fidelity DNA polymerase, Cat. #172-5302).

Gene *dmaW_Aj3* was prepared by PCR using *dmaW_Aj1* as template, and *dmaW_Aj2* was prepared by sequential extension PCR, using *dmaW_Aj3* as template, thus adding the second exon in two steps (Additional file 1: Table S1). The *easC_Aj* version without C-terminal PTS1 signal was prepared by PCR amplification of the coding sequence (CDS) without the 9 bps before the stop codon. The EasE_Aj N-terminal truncation (EasE_Aj -N sig.) was done by PCR, amplifying the corresponding CDS without the first 87 bps. The forward PCR primer inserted an alternative ATG translation start site. The fusion of an N-terminal signal peptide from Pdi1 to the N-truncated EasE_Aj was done by overlapping extension PCR, fusing the two amplicons to encode Pdi1-EasE_Aj (Additional file 1: Table S2). The CDS of the native yeast genes *pdi1* [GenBank:D00842], *ero1* [GenBank:NM_001182493], and *fad1* [GenBank:NM_001180104] were amplified from genomic DNA by PCR.

For expression, all genes were cloned into expression vectors based on pRS313, pRS315, and pRS316 [35]. These vectors had been provided with a new multi-cloning site (MCS) linker, inserted between the two *Pvu*II sites. The basic design of the MCS was *Srf*I-*Asc*I-*Bgl*II-*Hind*III-*Sfi*I(a)-*Sfi*I(b)-*Sac*II-*Sph*I-*Asc*I-*Srf*I [36]. The linker allowed cloning of promoter sequences into *Bgl*II and *Hind*III, and terminators into *Sac*II and *Sph*I restriction sites to create yeast expression cassettes. Promoters and terminators were amplified from yeast genomic DNA by PCR (Additional file 1: Table S3) for preparing three expression cassettes containing 1) a *GPD1* promoter and a *CYC1* terminator (G/C), 2) a *PGK1* promoter and an *ADH2* terminator (P/A), and 3) a *CUP1* promoter and an *ADH1* terminator (C/A). The new expression vectors were named pRS31X-G/C, pRS31X-P/A, and pRS31X-C/A, where X designates 3, 5, or 6. All genes used in this study (Table 1) were cloned into expression cassettes of these vectors.

### Construction and integration of yeast gene expression cassettes

Constructs for integration were prepared for the integration sites *YORWΔ22* and *YPRCΔ15* [37], and cloned in unique *EcoR*I and *Hind*III sites of a pUC19 vector backbone. The homologous regions were constructed from two PCR fragments, which were then combined by overlapping extension PCR. The PCR primers introduced the restriction sites *AscI* and *Not*I between the two original fragments, and *Sbf*I sites at the outer ends. The *KanMX* cassette, flanked by loxP sites, was excised from pUG6 [38] and inserted into *Not*I. Two expression cassettes (described above) were inserted into the *Asc*I site. The first cassette was amplified by PCR, changing one *Asc*I site to a *Mlu*I. After cloning this fragment, the second cassette was inserted into the single regenerated *Asc*I site. The entire construct was released by *Sbf*I and used for integration. This approach was used to first integrate *dmaW_Aj2* (G/C cassette) *and easF_Af* (P/A cassette) into the yeast genome at the *YORWΔ22* site. After excision of the *KanMX* marker [38], the *easC_Aj* (G/C cassette) and *easE_Aj* (P/A) cassette were integrated into the *YPRCΔ15* site. Yeast transformations were done using the LiAc method [39].

### Yeast strain and culture conditions

Chanoclavine-I production was achieved in several *S. cerevisiae* strain backgrounds, including S288C, W303, and others (not shown). The strain used to prepare the results in this report had the genotype *MATα*, *his3Δ1*, *leu2Δ0*, *lys2Δ0*, *ura3Δ0*. Engineered yeast strains were grown in standard SC broth with 2% glucose, minus leucine and histidine (ForMedium, Hunstanton, U.K.). When appropriate, CuSO_4_ was added to a final concentration of 300 μM for induction of gene expression. Cultures for analysis were started from re-streaked, single colonies. These were grown overnight in standard SC broth, and then diluted to an optical density, at 600 nm, of 0.1 to start the main culture. Main cultures were grown at 30°C with constant shaking at 150 rpm, 5 cm amplitude, for 72 hours in 250 ml shake flasks containing 25 ml medium. Final optical density was measured at 600 nm, typically reaching 12–14, although small variations were seen between individual experiments.

### Analytical procedures

For analysis yeast cultures were spun down for 10 min at 1000 × g. The pellet and the supernatant were separated. Without further purification, 5 μl of supernatant were injected in a UPLC-TOF (Waters Acquity™ Ultra Performance LC, Waters, Milford, Mass., USA) coupled to a micrOTOF-Q II (Bruker Daltonik GmbH, Bremen, Germany). Stationary phase was an Acquity UPLC® Bridged Ethyl Hybrid (BEH) C18, 1.7 μm, 2.1 × 100 mm column. Liquid chromatography used mobile phases of H_2_O + 0.1% formic acid (A), and acetonitrile + 0.1% formic acid (B), in a linear gradient of 1% to 100% B in 5 min. The column was washed for 1 min in 100% B, and then equilibrated for 1.5 min in 1% B. Detection of compounds was done by a photo diode array using the following parameters: λ range 210 nm to 500 nm. Resolution 1.2 nm. Sampling rate 5 points/s. ELSD parameters: gain 50, gas pressure 40 psi, nebulizer mode heating, power level 80%, drift tube 80°C. TOF parameters Source: End Plate Offset −500 V. Capillary −4500 V. Nebulizer 1.6 bar. Dry gas 8.0 l/min. Dry temperature 180°C. TOF parameters Scan mode: MS Scan. Mass range from 80 to 1000 m/z. Quantification of chanoclavine-I was based on in-house, purified reference compound.

### Preparative procedures

For compound purifications yeast cultures were spun down for 10 min at 1000 × g. The supernatant was adjusted to pH = 10 with 10 M NaOH and extracted by liquid/liquid extraction with an equal volume of ethyl acetate. The crude extract was dried under vacuum and reconstituted with dimethyl sulfoxide (DMSO) to a concentration of 100 mg/ml and then purified on a preparative HPLC system (Waters, Milford, Mass, USA). Stationary phase was an XBridge™ preparative C18, 5 μm, 19 × 250 mm column. Liquid chromatography used mobile phases of H_2_O + 0.1% trifluoroacetic acid (A), and acetonitrile + 0.1% trifluoroacetic acid (B), in a linear gradient of 1% to 30% B in 40 min. The column was washed for 5 min in 100% B, and then equilibrated for 5 min in 1% B. Fractions were collected every 2 min and analyzed as above. Fractions containing the purified analyte were pooled and dried under vacuum.

## Additional file

Additional file 1: Table S1 Primers for construction of dmaW variant. **Table S2.** N-terminal sequences of EasE_Aj and Pdi1_Sc. **Table S3.** Expression cassettes in pRS vectors. **Figure S1.** Alignment of *in silico* translated CDS predictions of DmaW enzymes. **Figure S2.** NMR data of Me-DMAT and chanoclavine-I. **Figure S3.** Alignment of EasE proteins, showing conserved cysteine residues. **Figure S4.** Alignment of EasE proteins with two BBE proteins, indicating putative FAD binding. **Figure S5.** Alignment of EasE protein sequences derived from GenBank.
