# Supplementary material for: The important ergot alkaloid intermediate chanoclavine-I produced in the yeast Saccharomyces cerevisiae by the combined action of EasC and EasE from Aspergillus japonicus
Source: Microb Cell Fact. 2014 Aug 12;13:95. doi: 10.1186/s12934-014-0095-2 (PMC4249865; doi:10.1186/s12934-014-0095-2)
Supplement: Additional file 1: Table S1 — Primers for construction of dmaW variant. Table S2. N-terminal sequences of EasE_Aj and Pdi1_Sc. Table S3. Expression cassettes in pRS vectors. Figure S1. Alignment of in silico translated CDS predictions of DmaW enzymes. Figure S2. NMR data of Me-DMAT and chanoclavine-I. Figure S3. Alignment of EasE proteins, showing conserved cysteine residues. Figure S4. Alignment of EasE proteins with two BBE proteins, indicating putative FAD binding. Figure S5. Alignment of EasE protein sequences derived from GenBank. [file 12934_2014_95_MOESM1_ESM.docx]

**Additional file 1**

**The key ergot alkaloid intermediate chanoclavine-I produced in yeast (*Saccharomyces cerevisiae*) by the combined action of EasC and EasE from *Aspergillus japonicus*.**

**Nielsen et al.; Microbial Cell Factories**

**Contains:**

Table S1: Primers for construction of dmaW variant

Table S2: N-terminal sequences of EasE_Aj and Pdi1_Sc

Table S3: Expression cassettes in pRS vectors

Figure S1: Alignment of *in silico* translated coding sequence predictions of DmaW enzymes

Figure S2: NMR data of Me-DMAT and chanoclavine-I

Figure S3: Alignment of EasE proteins, showing conserved cysteine residues

Figure S4: Alignment of EasE proteins with two BBE proteins, indicating putative FAD binding

Figure S5: Alignment of EasE protein sequences derived from GenBank

**Table S1** Primers for the construction of dmaW_Aj3 (1 & 2) using dmaW_Aj1 as template, and of dmaW_Aj2 (1 & 3, followed by 1 & 4) using dmaW_Aj3 as template.

__________________________________________________________________________________________

1. ACATAAGCTTAAAATGACTGCTGGTCAAGGTAT
2. ACATCCGCGGTCAGGTAACCCAATCACCAGTTTC
3. GGTCTTGGTCAGCTTGGACAGCTCTTGGACGGTGGCTGAAAGGCCAGGGTCCACATGGACAGAGTTGAAGGATGAGGTAACCCAATCACCAGTTTC
4. ATGTCCGCGGCTAGTACTGGGTGATGACGCCAGGCTCTGATCCATCTGGGGTTAGTGGTAGTTTTGTCTCCCTAACGGTGGTTCCGGCGGTCTTGGTCAGCTTGGAC

**____________________________________________________________________________________________________**

**Table S2** N-terminal sequences of the wt EasE_Aj (top) and the fusion protein Pdi1-EasE_Aj with the Pdi1 signal peptide (bottom)

____________________________________________________________________________________________________

Protein N-terminal sequence

EasE_Aj MGQSRGILGGVRQLILVILVGAYLSRLSAVD**DDRHDCRCRPGEP…**

Pdi1-EasE_Aj MKFSAGAVLSWSSLLLASSVFAQQ**DDRHDCRCRPGEP…**

____________________________________________________________________________________________________

The predicted wt EasE_Aj N-terminal signal sequence was deleted to create a truncated version (EasE_Aj -N sig.) starting at DDR… (in bold). This truncated version was used to construct the fusion protein (Pdi1-EasE_Aj), in which the N-terminal sequence from Pdi1 (underlined) replaces the wt EasE_Aj N-terminus. Both constructs were prepared by PCR and confirmed by sequencing.

**Table S3** Cassettes for cloning and expressing genes were constructed by PCR amplification of native yeast (*S. cerevisiae*) promoters and terminators. The number of bases upstream or downstream of the corresponding CDS is listed in parenthesis.

__________________________________________________________________________________________

Name Promoter (bps) Terminator (bps)

G/C cassette Gpd1 (680) Cyc1 (320)

P/A cassette Pgk1 (750) Adh2 (300)

C/A cassette Cup1 (450) Adh1 (345)

____________________________________________________________________________________________________


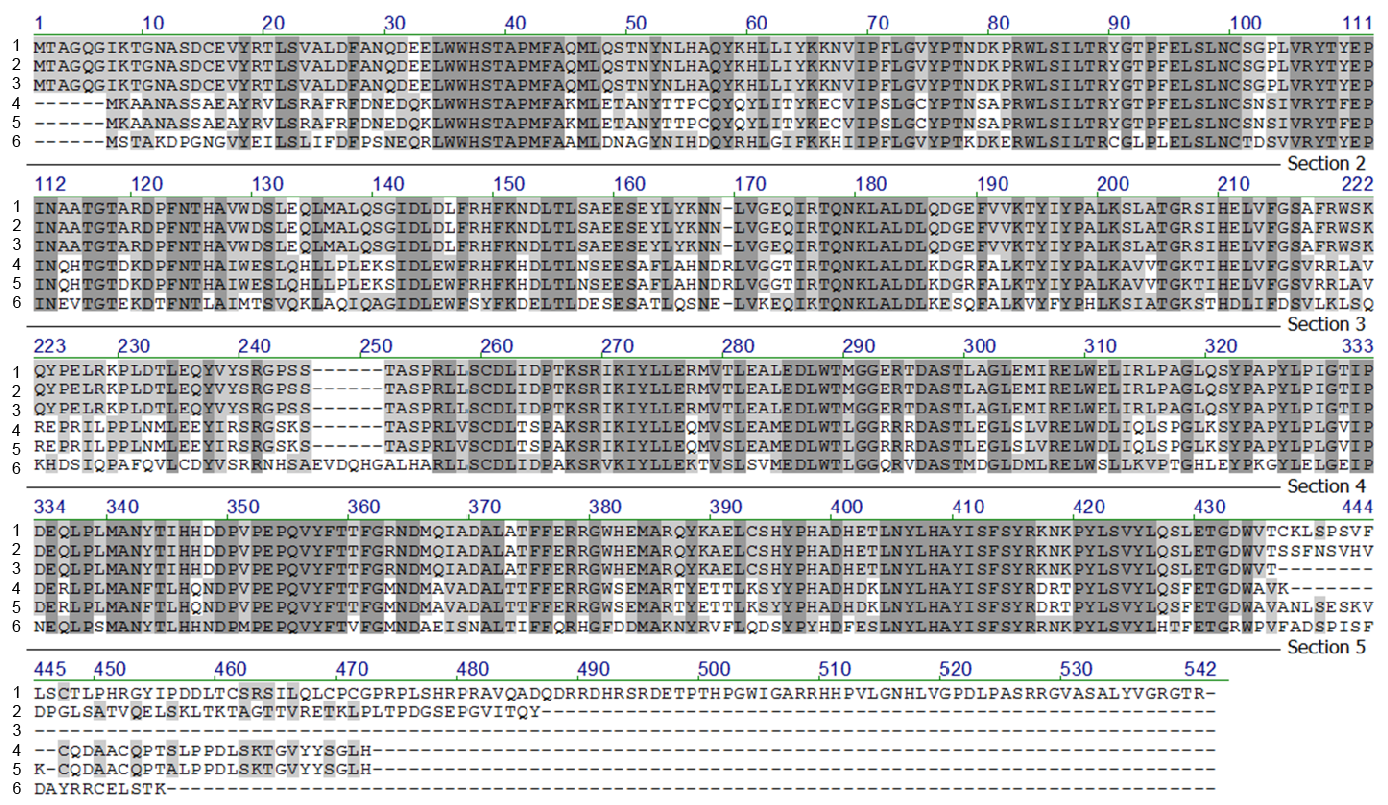


**Figure S1** Alignment of *in silico* translated coding sequence predictions of DmaW from *A. japonicus*: DmaW_Aj1 (**1**), DmaW_Aj2 (**2**), and DmaW_Aj3 (**3**) all derived from WO2012/116935 A2, with DmaW_Af1 (**4**) derived from XM_751048 (*A. fumigatus*), DmaW_Af2 (**5**) derived from AY775787 (*A. fumigatus*), and DmaW_Cp (**6**) derived from AJ011963 (*C. purpurea*). A high degree of conservation is seen, except for at the C-terminal end. Note also that DmaW_Af1 (used in this study) has an 8 amino acid “deletion” compared to DmaW_Af2, the latter of which was used in a previous study [6].

**
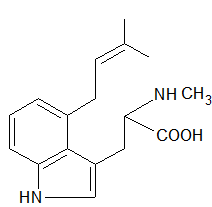
**

**Figure S2a** NMR data of purified Me-DMAT eluted at 7.9 min.: ^1^H NMR (600 MHz, DMSO-d6) δ ppm 1.61 (s, 3 H) 1.63 (s, 3 H) 2.45 (s, 3 H) 3.19 (dd, J=16.28, 8.33 Hz, 1 H) 3.42 (dd, J=16.00, 4.94 Hz, 1H) 3.57 - 3.63 (m, 3 H) 5.13 - 5.21 (m, 1 H) 6.74 (d, J=7.15 Hz, 1 H) 6.98 (t, J=7.50 Hz, 1 H) 7.14 (s, 1H) 7.16 - 7.21 (m, 1 H)


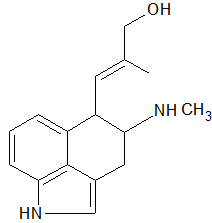


**Figure S2b** NMR data of purified chanoclavine-I, eluted at 4.3 min. +/- 0.2 min: ^1^H NMR (600 MHz, *DMSO-d*_6_) δ ppm 1.82 (s, 3 H) 2.67 (t, *J*=5.22 Hz, 3 H) 3.11 (dd, *J*=15.65, 7.79 Hz, 1 H) 3.32 (dd, *J*=15.58, 4.00 Hz, 1 H) 3.54 (m, *J*=7.20, 3.60, 3.60 Hz, 1 H) 3.95 (s, 2 H) 4.25 (dd, *J*=9.39, 7.60 Hz, 1 H) 5.36 (dd, *J*=9.74, 0.89 Hz, 1 H) 6.71 (d, *J*=7.11 Hz, 1 H) 7.08 (t, *J*=7.60 Hz, 1 H) 7.16 (s, 1 H) 7.25 (d, *J*=8.09 Hz, 1 H) 8.48 (br. s., 1 H) 8.73 (br. s., 1 H) 11.01 (s, 1 H)


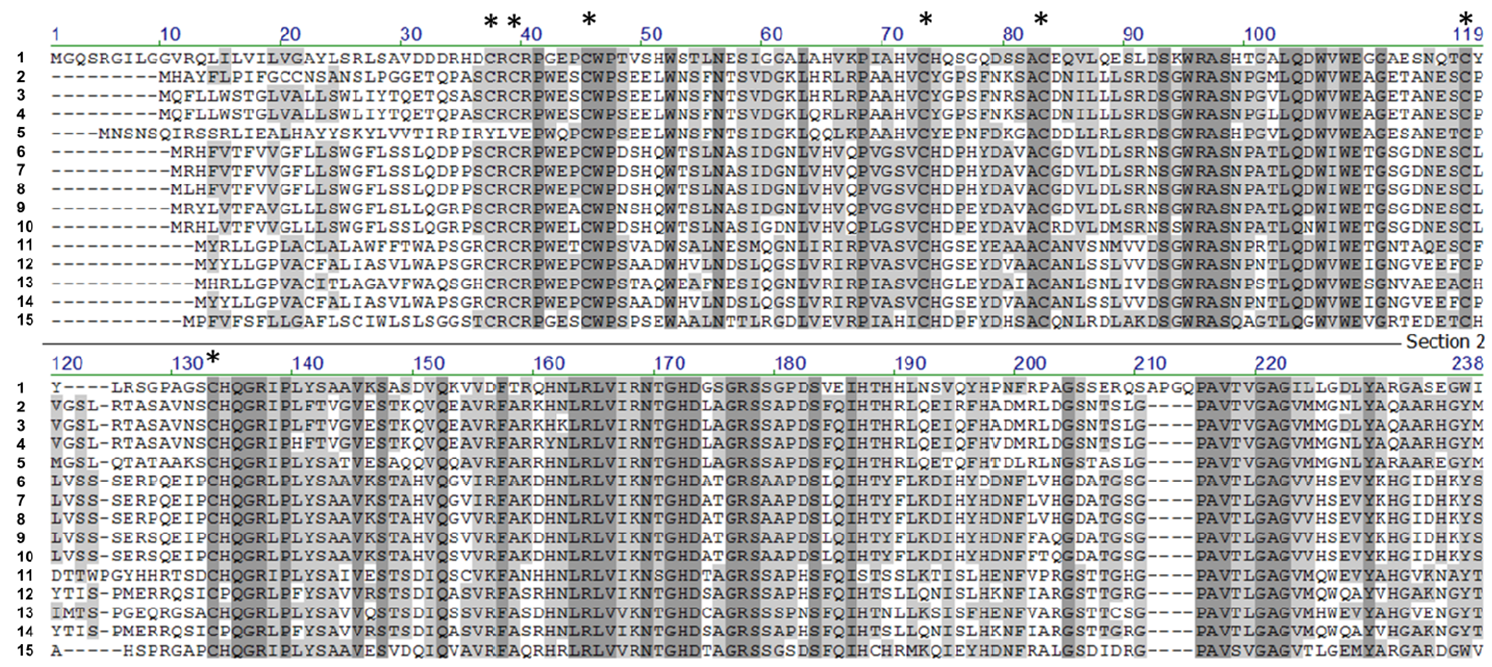


**Figure S3** Protein sequence alignment (N-terminal part) of EasE_Aj predicted from WO2012/116935 A2 (1) with close homologues from GenBank (2-13), including EasE_Cp based on the new JN186799 (14) and EasE_Af based on our new coding sequence prediction of NC_007195 (15). The alignment shows a high degree of conservation past the predicted N-terminal signal peptide, also in the new EasE_Cp and EasE_Af. In particular, the cysteines in this region are highly conserved (indicated by asterisks). GenBank accession numbers used here (2-13) are: EGE00007 (*Trichophyton tonsurans*); XP_003024000 (*Trichophyton verrucosum*); XP_003233054 (*Trichophyton rubrum*); XP_002846185 (*Arthroderma otae*); ABM91450 (*Neotyphodium lolii*); AET10036 (*Epichloe festucae*); AEV21233 (*Epichloe typhina*); AET10059 (*Epichloe brachyelytri*); AET10053 (*Epichloe glyceriae*); ABV57823 (*Claviceps fusiformis*); AET79192 (*Claviceps purpurea*); and AEV21224 (*Periglandula ipomoeae*).


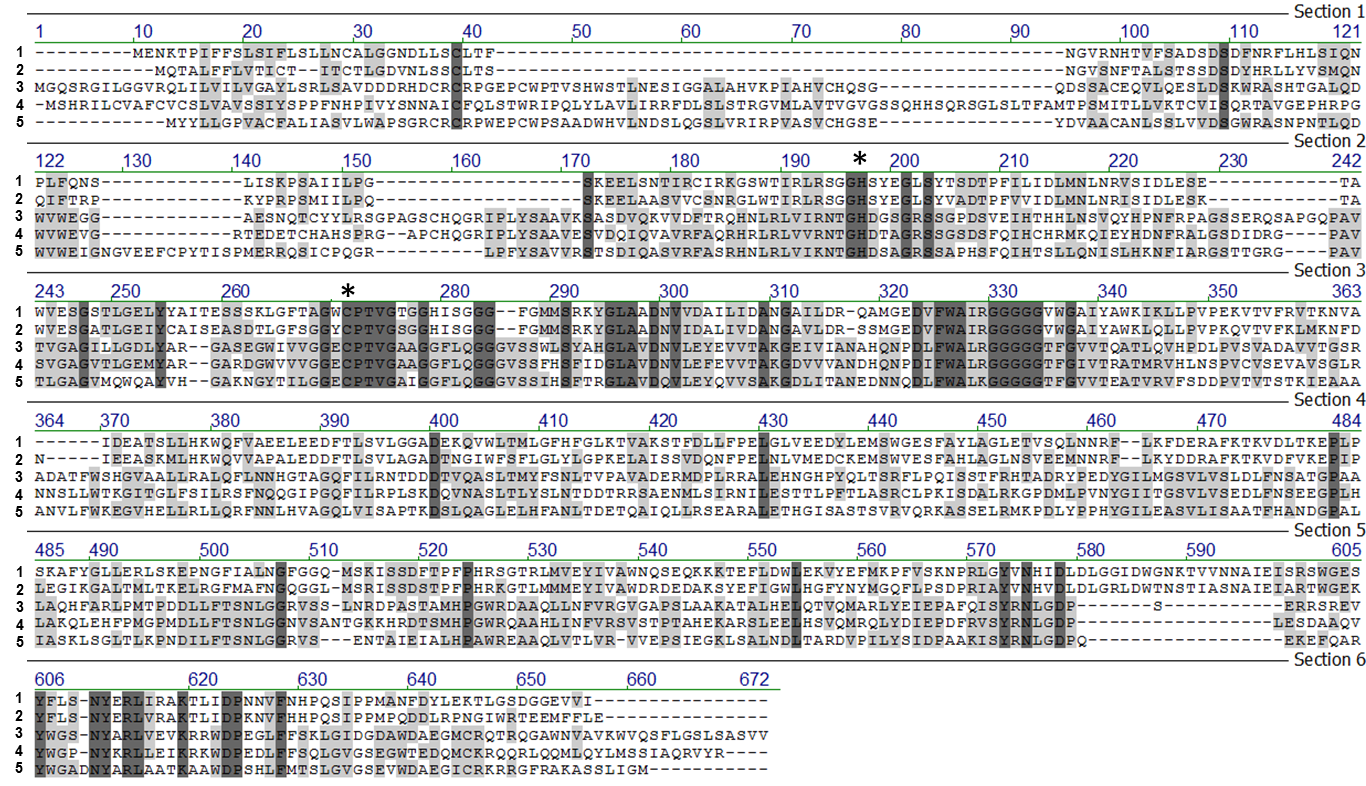


**Figure S4** Alignment of BBE proteins from *Eschscholzia californica* (1) and *Berberis stolonifera* (2) with EasE_Aj from *A. japonicus* (3), EasE_Af from *A. fumigatus* (4), and EasE_Cp from *C. purpurea* (5). The predicted FAD binding domain is conserved in the two classes of proteins. The histidine and cysteine involved in the bi-covalently binding of FAD, see reference [27], are indicated by asterisks (*).


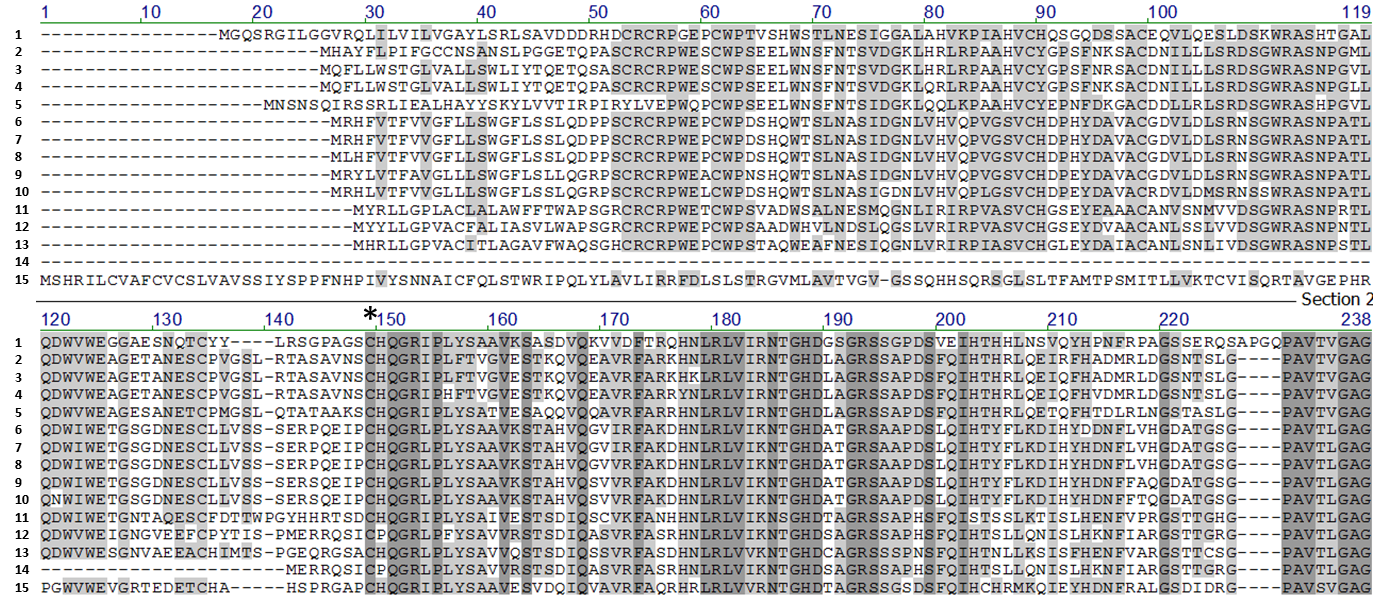


**Figure S5** Protein sequence alignment (N-terminal part) of EasE_Aj predicted from WO2012/116935 A2 (1) with close homologues from GenBank (2-13), including EasE_Cp based on AJ011965 (14) and EasE_Af based on XM_751049 (15). The alignment shows complete lack of the conserved N-terminal domain in EasE_Cp (14) and a lack of homology in EasE_Af (15) to this region (upstream of asterisk), which may explain our observed lack of functional expression of these two genes. GenBank accession numbers used here (2-13) are: EGE00007 (Trichophyton tonsurans); XP_003024000 (Trichophyton verrucosum); XP_003233054 (Trichophyton rubrum); XP_002846185 (Arthroderma otae); ABM91450 (Neotyphodium lolii); AET10036 (Epichloe festucae); AEV21233 (Epichloe typhina); AET10059 (Epichloe brachyelytri); AET10053 (Epichloe glyceriae); ABV57823 (Claviceps fusiformis); AET79192 (Claviceps purpurea); and AEV21224 (Periglandula ipomoeae).
